# Prognostic potential of radiomics evaluation of lung artery thrombus for pulmonary embolism patients

**DOI:** 10.1007/s11548-025-03530-x

**Published:** 2025-10-22

**Authors:** Lea Ehrhardt, Patrique Fiedler, Alexey Surov, Sylvia Saalfeld

**Affiliations:** 1 Institute of Biomedical Engineering and Informatics, Technische Universität Ilmenau, Gustav-Kirchhoff-Str. 2, 98693 Ilmenau, Thuringia Germany; 2 Department of Radiology, Neuroradiology and Nuclear Medicine, Johannes Wesling University Hospital, Ruhr University Bochum, Hans-Nolte-Str. 1, 32429 Minden, North Rhine-Westphalia Germany; 3 Department of Medical Informatics and Statistics, University Hospital Schleswig-Holstein, Campus Kiel, Arnold-Heller-Str. 3, 24118 Kiel, Schleswig-Holstein Germany

**Keywords:** Radiomics, Pulmonary embolism, Thrombus, Troponin, Mortality, Classification

## Abstract

**Purpose:**

This study evaluates radiomics correlation with mortality and suitability as prognostic indicator for troponin for pulmonary embolism to enhance prognostic accuracy and guide personalized treatment strategies with the help of machine learning. We conducted an initial study focusing on texture information of the arterial thrombus.

**Method:**

Computed tomography (CT) of the lung from 86 patients with pulmonary embolism was used. As target variables, we used patients 30-day mortality and troponin results. Each arterial thrombus was manually segmented. After the extraction of their radiomics features and the reduction via correlation analysis and 12 feature selection methods, these and the target variables were given to 12 different classification methods to record the accuracies (Acc.), F1-scores (F1) and ROC curve areas under the curve (AUC) for comparison and evaluation.

**Results:**

The resulting accuracy achieved was 0.967, the F1-score 0.973 for class 0 and 0.967 for class 1 and the AUC around 0.9686. The feature selection methods which resulted in the highest results were ReliefF (RF), Logistic Regression (LOR) and CART Classification (CARTC). For the classification methods, Support Vector Machines (SVM), eXtreme Gradiant Boosting (XGB) and Ensemble Bagged Trees (EBT) lead to the highest results. Firstorder, Shape and gray-level co-occurrence matrix (GLCM) were the most selected radiomics feature classes.

**Conclusion:**

Within this study, we conducted radiomics feature extraction within a medical image data analysis pipeline with subsequent correlation analysis and training of classifiers for patients with pulmonary lung embolism. We could show that the radiomics features correlated with patient’s morphology as well as troponin range with an accuracy of 0.967 and 0.9302, respectively, yield high potential for prognosis and treatment strategy of pulmonary embolism patients in the future.

## Introduction

Acute pulmonary embolism (APE) is one of the most occuring cardiovascular diseases and shows a high mortality rate [1]. A fast and accurate diagnosis is therefore of importance. In the clinical routine, CT pulmonary angiogram (CTPA) is used as a standard device for the image acquisition of APE and its resulting parameters can be utilized to predict medical outcomes like 30-day mortality. This can be derived for example from the information about the right ventricle enlargement [2] and inferior vena cava (IVC) reflux [3], which was proved to also correlate with tricuspid regurgitation and levels of troponin [4, 5], which is a globular protein that can be used to access short- and long-term risks of APE patients mortality. These diagnostic indicators are derived from the recorded medical image, which leads to the assumption that image analysis could further help with APE diagnosis and assessment.

Other studies explored these relations between image features and APE while focusing on the embolus area. Jakob Leonhardi et al. have shown that texture information of CTPA images of pulmonary embolism also correlates with different patient blood markers and outcomes, like the 30-day mortality [6]. Prediction of patients’ survival being feasible can also be found in different papers looking into the implementation of texture features for prediction tasks. Jennifer Gotta et al. demonstrated that these features can be used in a classification process to distinguish between healthy and APE patients as well as to predict the overall survival [7], whereas Dawei Wang et al. looked specifically into the comparison of good and poor prognosis of APE patients [8]. Both research articles conclude that texture features of the embolus area can be used for different prediction tasks regarding APE analysis. This pathology is therefore an important region for accessing the severity of APE.

One way to extract the image features for different applications like classification tasks is the radiomics feature extraction. In recent years radiomics has emerged as a promising field within medical imaging, as a quantitative feature extraction approach for radiographic images for a comprehensive analysis. The integration of radiomics with tomographic image data of cancer patients holds immense potential for personalized medicine and prediction of patient outcome [9, 10]. But also for other medical conditions and diseases radiomics shows advancing concepts to improve medical diagnosis and the planning of treatment and therapy.

The focus of this work was therefore to look for possible correlation between radiomics features of the thrombus area, 2D and 3D representation, of patients with pulmonary embolism and the mortality as well as troponin, which was proved to be connected to the severity of APE [5], but was not yet addressed directly in the radiomics context. We further wanted to evaluate the possibility of radiomics feature-based classifications to predict these two clinical outcome variables.

## Materials and methods

In this section, we will discuss the utilized materials and methods regarding the medical image data acquisition, preparation, and segmentation as well as the radiomics feature extraction and analysis.

### Medical image dataset

The present dataset is part of a retrospective study that was approved by the institutional review board (Nr. 145/21, Ethics Committee, Otto-von-Guericke University of Magdeburg, Magdeburg, Germany).

From the original study (2015–2021), 86 CT scans were used for the present analysis, and for each of these manual segmentation was conducted. 58 male and 28 female patients with mean age of 64.7 ± 14.8 were analyzed. Patients with chronic PE were excluded. The determination of chronic and acute pulmonary embolism followed the general guidelines [11, 12]. Scores such as Pulmonary Embolism Severity Index (PESI), simplified Pulmonary Embolism Severity Index (sPESI) and Geneva score were used for the specification. A CTPA was conducted for the patients before thrombulytic treatment was administered. This treatment was also not applied during the recordings. During the follow-up for 30 days, the patients’ mortality and other parameters were accessed [13]. The mortality was later used as one of the target variables.

The image acquisition was performed on multi-slice CT scanners (Siemens Somatom Definition AS+, Siemens Healthcare, Germany or Canon Aquilion Prime, Canon Medical Systems, Ottawara, Japan). An iodinated contrast agent (60–150 ml Accupaque 300 mg/ml, GE Healthcare Buchler GmbH & Co.KG, Braunschweig, Germany, or Imeron 300, Bracco Imaging Deutschland GmbH, Konstanz, Germany) was intravenously administrated via peripheral venous line at rate of 3.0$-$4.0 ml/s for all cases. In the pulmonary trunk, an automatic bolus tracking was further applied with a trigger of 100 Hounsfield units (HU). The imaging parameters in general were: 100–120 kVp, 25–200 mAs (tube current modulated 50–400 mA), slice thickness and slice interval of 1 mm and pitch factor of 1.4 [13].

### Methods

Our general pipeline consists of the following steps: manual segmentation of the thrombus area with MeVisLab3.1 VS2017-64 (Fraunhofer MEVIS and MeVis Medical Solutions AG, Germany; https://www.mevislab.de), extraction and calculation as well as analysis of the PyRadiomics results with the programming framework Spyder 3.11 (Anaconda Inc., USA; https://www.spyder-ide.org/) and storing of the results with Excel Ver. Office 16 (Microsoft Corporation, USA; https://www.microsoft.com/excel).

**Fig. 1:**
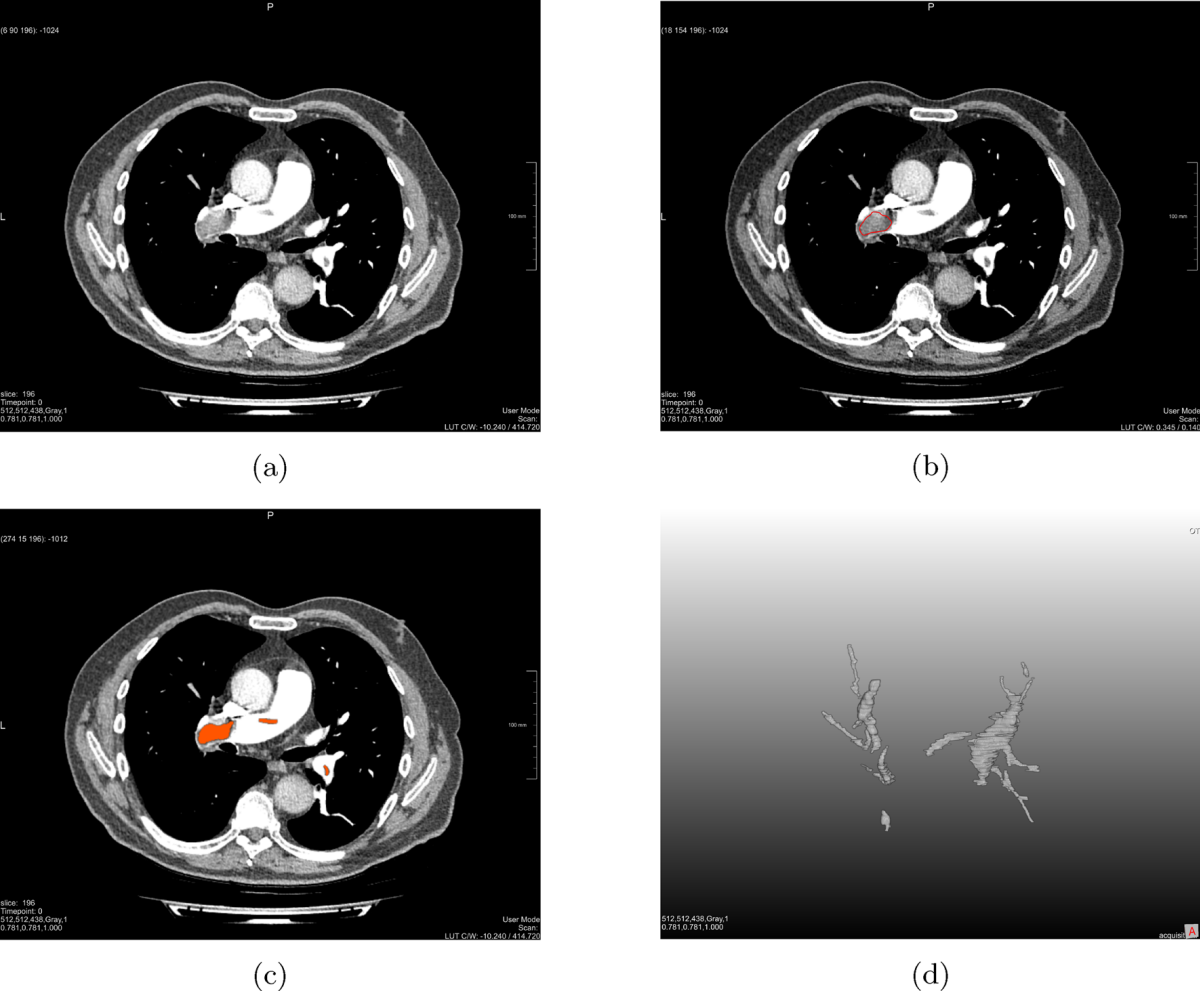
Illustration of the manual segmentation of the thrombus area of a patient. **a** Patient unprocessed CT image. **b** CT slice with one drawn mask illustrated as a red line. **c** CT image with segmented masks for the shown slice illustrated as red areas. **d** Resulted segmented thrombus as 3D voxel masks

The segmentation masks were manually segmented via MeVisLab [14] and verified by a radiologist before being converted to be used in Python for the radiomics analysis. Figure 1 represents the segmentation process in MeVisLab for one patient CT image.

To ensure that we were able to extract radiomics features from the manually segmented masks, we included masks with at least 0.5 cm$^3$ volume. With this predefinition, all masks could be used for the radiomics analysis without any complications.

We segmented 2D masks and converted them to 3D voxel masks for two different studies. The mortality was selected to represent the first target variable. For the second target variable, we converted the troponin parameter values based on their median resulting in binary target variables. The median was used because our data for the troponin value were not normally distributed. The data were further balanced by using a combination of over- and undersampling in MATLAB 2023b (The MathWorks, Natick, MA, USA; www.mathworks.com) [15]. This pre-processing step was performed, because the data exhibited a much larger majority than minority class.

We included 5 k-fold cross-validation for both target variables independently resulting in two runs $run_\mathrm{{{mort}}}$ and $run_\mathrm{{{trop}}}$. A total of 369 features were extracted. These were reduced to 10 features during the correlation analysis followed by a feature selection process.

### Radiomics workflow

In the following, the PyRadiomics workflow will be listed and explained.

The DICOM CT data of each patient were loaded, and the radiomics features were extracted in Python via the PyRadiomics function [16]. 86 medical image data were used for the extraction of both runs ($run_\mathrm{{{mort}}}$ and $run_\mathrm{{{trop}}}$). PyRadiomics extracts multiple feature classes which are defined as Shape (2D and 3D), Firstorder, gray-level co-occurrence matrix (GLCM), gray-level run length matrix (GLRLM), gray-level size zone matrix (GLSZM), gray-level dependence matrix (GLDM) and neighborhood gray tone difference matrix (NGTDM). More information about these classes can be found on the official website [16]. Shape features were excluded for the 2D masks, but retained for the 3D due to 2D shape being negligible for the analysis though important for the 3D volume calculations.

We applied ZScoring to the feature extraction results in Python and stored the adjusted data as an Excel table. Correlation analysis was applied to remove correlating features (Pearson correlation coefficient > 0.85) within Python [17]. Followed by a k-fold cross-validation, the data were split into 5 training and test sets. The feature reduction and classification methods were calculated for each of the 5 folds.

A total of 12 feature reduction methods were applied to find the most significant features. For our analysis, we decided to decrease them to 10 to reduce dimensionality and avoid further correlation between features which reduced computational effort of the analysis as well. The reduction count of 10 was chosen after testing multiple other factors. The feature reduction methods which were used consist of ReliefF (RF) [18], Analysis of Variance (ANOVA), Chi-squared (Chi2), Linear Regression (LIR), Logistic Regression (LOR), CART Regression (CARTR), CART Classification (CARTC), Random Forest Regression (RFR), Random Forest Classification (RFC) [19], FisherScore (Fisher), Laplacian [20] and GradientBoost (RB) [18]. Dictonaries were created for training, test sets, feature names and positions. Different papers list these methods as suitable for the reduction in radiomics features, which is why they were used for our calculations [21–23].

For each feature reduction method, 12 classification models were trained to perform prediction calculation. The utilized classification methods include Neural Networks (NN), Decision Tree (DTREE), Support Vector Machines (SVM), K-Nearest Neighbor (KNN), Random Forest (RF), Logistic Regression (LRC), Gaussian Naive Bayes (GNB) [19], eXtreme Gradient Boosting (XGB) [18], Stochastic Gradient Descent (SGD), Bernoulli Naive Bayesian (BNB), Ensemble Bagged Trees (EBT) and AdaBoost (ABC) [19]. These classification methods were chosen after researching different papers regarding radiomics analysis for medical pathologies. The used networks were applied to predict different medical outcomes [9, 10, 22, 24–26].

After the calculation and analysis of the k-folds, the predictions of the accuracies per fold were combined and a confusion matrix calculation was applied on them. As a result, we were getting an overall accuracy, ROC AUC and F1-score additionally.

All calculated scores as well as the best feature reduction and classification methods together with the reduced features were all stored accordingly.

**Fig. 2:**
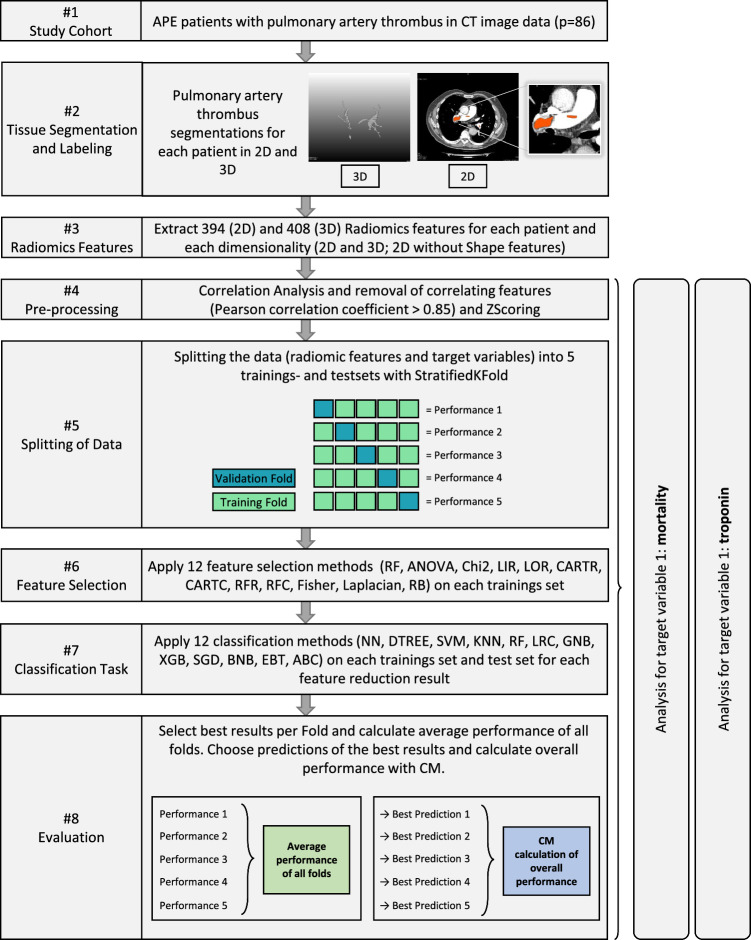
Applied process of radiomics feature extraction, preparation of data and radiomics analysis

This pipeline is displayed in Fig. 2.

For a later comparison, the predictive power of the right/left ventricular (RV/LV) ratio for mortality and troponin was calculated additionally. For this, 11 of the previously used classification methods, DTREE excluded, were applied and the accuracy, F1-score and ROC curve AUC were also calculated. Like before, a 5 k-fold cross-validation was used and two additional runs to check for consistency of the analysis were done.

**Table 1:** Overview about the highest accuracies (Acc.), F1-scores (F1) and ROC curve AUCs

	2D			3D		
	Acc.	F1	ROC AUC	Acc.	F1	ROC AUC
$$run_\mathrm{{{mort}}}$$						
Run 1	0.9419	0.95/0.94	0.9405	0.9651	0.97/0.96	0.9648
Run 2	0.9419	0.95/0.94	0.9405	0.9767	0.98/0.98	0.9762
Run 3	0.9419	0.95/0.94	0.9405	0.9651	0.97/0.96	0.9648
**Mean**	0.9419	0.945	0.9405	0.96899	0.97	0.9686
$$run_\mathrm{{{trop}}}$$						
Run 1	0.9302	0.93/0.93	0.9307	0.9302	0.93/0.93	0.9318
Run 2	0.9302	0.93/0.93	0.9307	0.90698	0.91/0.9	0.9091
Run 3	0.9302	0.93/0.93	0.9307	0.8953	0.9/0.89	0.8966
**Mean**	0.9302	0.93	0.9307	0.9109	0.91	0.9125

Shown are the results of the three code iterations (Run1–3) of the 2D and 3D masks for the analysis with the mortality ($run_\mathrm{{{mort}}}$) and troponin ($run_\mathrm{{{trop}}}$) as the target variable. The values were calculated with the best predictions of the code run compared to the ground truth

## Results

The resulting overall accuracies for the 2D masks are for $run_\mathrm{{{mort}}}$ 0.9419 and the F1-score 0.95 for class 0 and 0.94 for class 1 with the ROC Curve AUC 0.9405. For $run_\mathrm{{{trop}}}$, respectively, the accuracies are 0.9302, the F1-score 0.93 for both classes and the ROC Curve AUC 0.9307.

In the 3D runs, we are getting the following results: For $run_\mathrm{{{mort}}}$, the accuracies are 0.969, the F1-score 0.973 for class 0 and 0.967 for class 1 and the AUC 0.9686. We obtain for $run_\mathrm{{{trop}}}$ accuracies 0.9109, F1-scores 0.913 for class 0 and 0.9067 for class 1 and AUCs 0.9125.

The results are listed in Table 1.

To verify the reproducibility of the results, 2 additional code runs (Run 2 and Run 3) were performed. The standard deviation is calculated to see if the radiomics analysis stays stable over these repetition runs. We are getting the following findings: For the $run_\mathrm{{{mort}}}$ data of the 2D masks, we are getting for the accuracy as well as for the F1-score and AUC a standard deviation of < 0.0001. We see the same standard deviation of < 0.0001 for $run_\mathrm{{{trop}}}$.

The standard deviation for the 3D masks is in $run_\mathrm{{{mort}}}$ for the accuracy 0.0067, for the F1-score 0.0087, and for the AUC 0.0066. During the $run_\mathrm{{{trop}}}$, the accuracy presents a standard deviation of 0.0178, the F1-scores of 0.018 and the AUC of 0.0178.

**Fig. 3:**
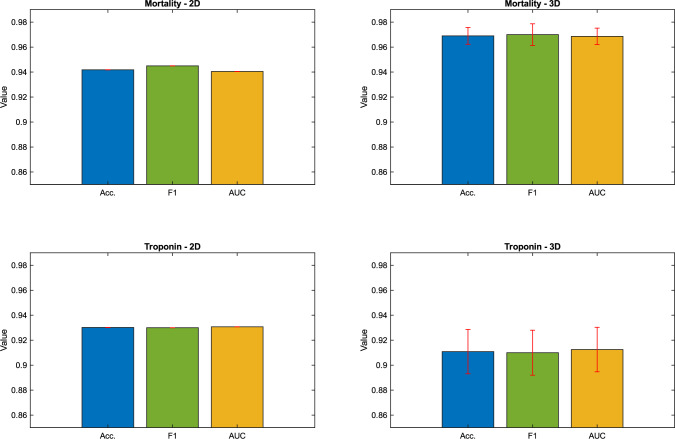
Barplots of the analysis of 2D and 3D masks with mortality and troponin as the target variable ($run_\mathrm{{{mort}}}$ and $run_\mathrm{{{trop}}}$). Shown are the visualizations of the accuracy (Acc.) in blue, F1-score (F1) in green and ROC curve AUC in yellow/orange. The red lines are the standard deviations of the metrics respectively

Figure 3 shows the barplot visualizations comparing $run_\mathrm{{{mort}}}$ and $run_\mathrm{{{trop}}}$ in the 2D and 3D run.

**Fig. 4:**
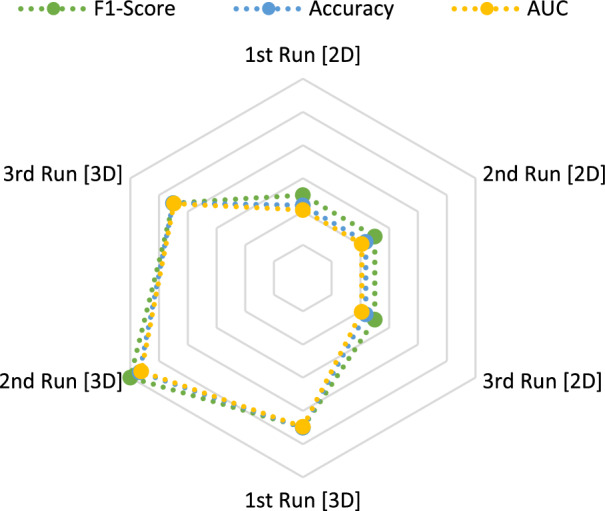
Radar chart to compare the calculated metrics for $run_\mathrm{{{mort}}}$. Blue = accuracy. Green = F1-score. Yellow/orange = ROC curve AUC

**Fig. 5:**
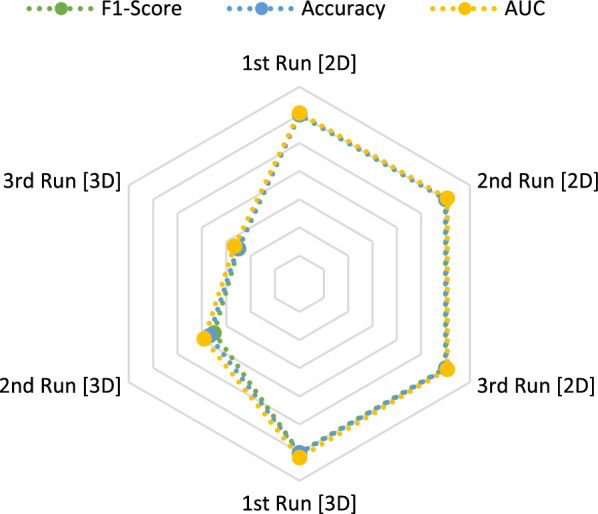
Radar chart to compare the calculated metrics for $run_\mathrm{{{trop}}}$. Blue = accuracy. Green = F1-score. Yellow/orange = ROC curve AUC

**Radar charts** represent a method to compare metrics of different models based on how well these models are performing. Figures 4 and 5 compare the performance of the accuracy, F1-score and AUC of $run_\mathrm{{{mort}}}$ and $run_\mathrm{{{trop}}}$ in all 2D and 3D runs.

**Classification methods in relation:**

**Fig. 6:**
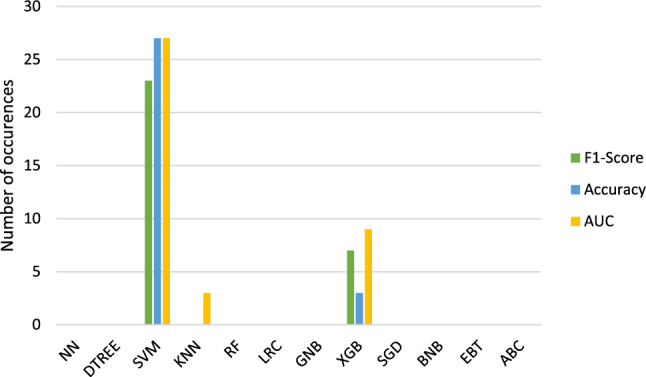
Classifications with highest resulting accuracies, F1-scores and AUCs for $run\_\mathrm{{{mort}}}$. Blue = accuracy. Green = F1-score. Yellow/orange = ROC curve AUC

**Fig. 7:**
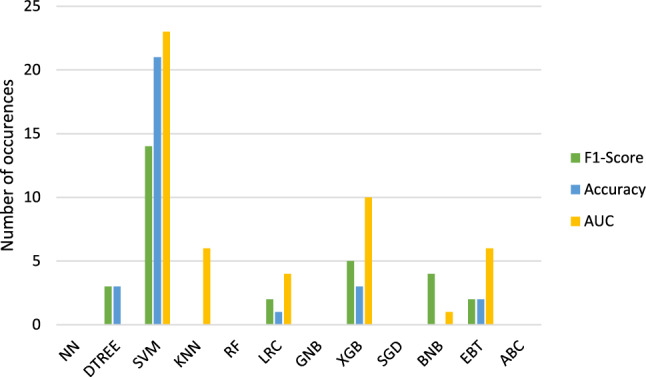
Classifications with highest resulting accuracies, F1-scores and AUCs for $run\_\mathrm{{{trop}}}$. Blue = accuracy. Green = F1-score. Yellow/orange = ROC curve AUC

Figures 6 and 7 display the classification methods which lead to the highest results in accuracy, F1-score and AUC.

Table 2 shows the classification methods which resulted the most often in the highest metrics.

The methods which resulted in the highest scores in general ($run_\mathrm{{{mort}}}$ and $run_\mathrm{{{trop}}}$) are therefore **SVM, XGB and EBT** followed by **KNN and LRC**.

**Table 2:** Overview about the classification methods which lead to the highest accuracies (Acc.), F1-scores (F1) and ROC curve AUCs (AUC) for the two target variable mortality ($run_\mathrm{{{mort}}}$) and troponin ($run_\mathrm{{{trop}}}$)

	$run_\mathrm{{{mort}}}$	$run_\mathrm{{{trop}}}$
Acc.	SVM	SVM XGB
	XGB	DTREE EBT
		LRC
F1	SVM	SVM XGB
	XGB	BNB DTREE
		LRC EBT
AUC	SVM	SVM XGB
	XGB	KNN EBT
	KNN	LRC
Overall	SVM	SVM XGB
	XGB	EBT LRC
	KNN	DTREE KNN

**Comparing Feature Selection Methods:**

**Fig. 8:**
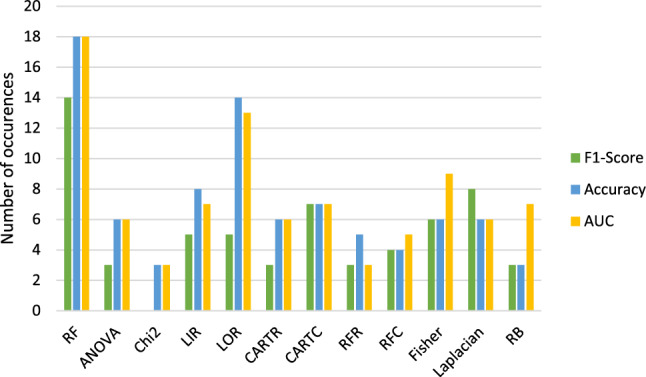
Combined bar graph about how often the feature selection methods resulted into the highest accuracies, F1-scores and AUCs for $run\_\mathrm{{{mort}}}$. Blue = accuracy. Green = F1-score. Yellow = ROC curve AUC

**Fig. 9:**
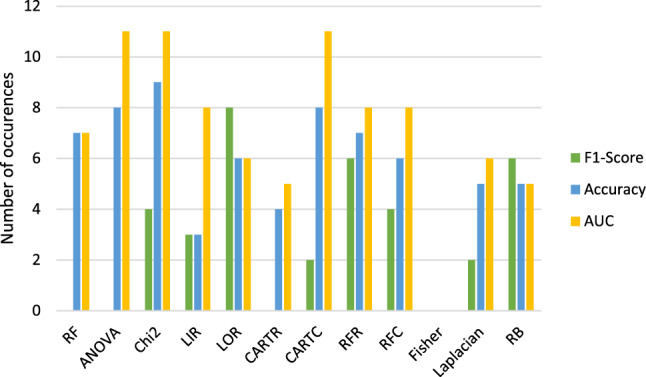
Combined bar graph about how often the feature selection methods resulted into the highest accuracies, F1-scores and AUCs for $run\_\mathrm{{{trop}}}$. Blue = accuracy. Green = F1-score. Yellow = ROC curve AUC

Figures 8 and 9 compare the feature reduction methods of $run_\mathrm{{{mort}}}$ and $run_\mathrm{{{trop}}}$ which lead to the highest results in accuracy, F1-score and AUC.

Table 3 shows the feature reduction methods which resulted the most often in the highest metrics.

**Table 3:** Overview about the feature selection methods which lead to the highest accuracies (Acc.), F1-scores (F1) and ROC curve AUCs (AUC) for the two target variable mortality ($run_\mathrm{{{mort}}}$) and troponin ($run_\mathrm{{{trop}}}$)

	$run_\mathrm{{{mort}}}$	$run_\mathrm{{{trop}}}$
Acc.	RF LOR	Chi2 ANOVA
	LIR	CARTC RF
	CARTC	RFR
F1	RF Laplacian	LOR RFR
	CARTC	RB Chi2
	Fisher	RFC
AUC	RF LOR	ANOVA Chi2
	Fisher LIR	CARTC LIR
	CARTC RB	RFR RFC
Overall	RF LOR	Chi2 CARTC
	CARTC Fisher	RFR LOR
	LIR Laplacian	ANOVA

Therefore, the method which resulted in the highest scores in general ($run_\mathrm{{{mort}}}$ and $run_\mathrm{{{trop}}}$) is **RF, LOR and CARTC** followed by **ANOVA and LIR**.

**Outcome of the most selected Radiomics Features:**

**Fig. 10:**
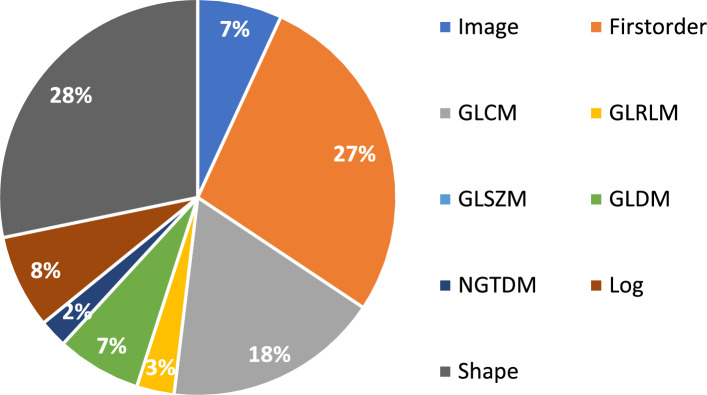
Overview about most important feature families during our calculations in percentage

In general, mostly **Shape, Firstorder and GLCM** features are selected as the most significant radiomics features. A percentage overview is shown in Fig. 10.

The most significant radiomics features overall are (from most significant to least): Image-Mean, firstorder_InterquartileRange, firstorder_10Percentile, firstorder_Entropy, glcm_ClusterShade, shape_Maximum2DDiameterRow, shape_LeastAxisLength and shape_Maximum2DDiameterColumn.

**Fig. 11:**
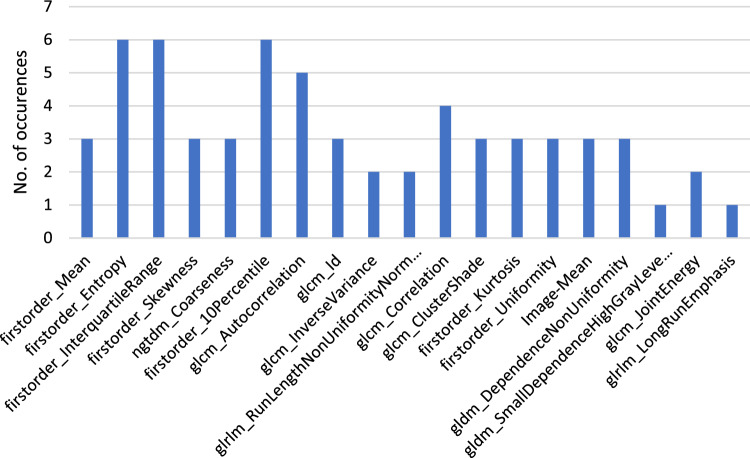
Most significant features of $run_\mathrm{{{mort}}}$ and $run_\mathrm{{{trop}}}$ of the 2D masks in a bar diagram overview

**Fig. 12:**
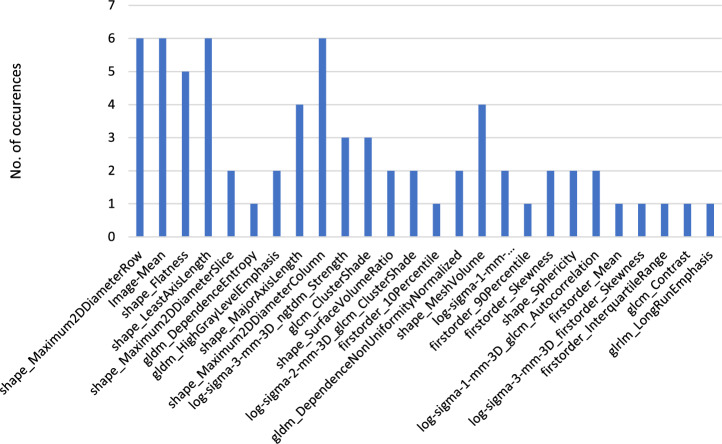
Most significant features of $run_\mathrm{{{mort}}}$ and $run_\mathrm{{{trop}}}$ of the 3D masks in a bar diagram overview

A visualization of these features divided by 2D and 3D masks is shown in Figs. 11 and 12.

**RV/LV ratio predictive power**

The classification with the RV/LV ratio was able to achieve accuracies of 0.783 for the mortality and 0.6418 for troponin. The F1-scores were around 0.765 and 0.64 and the AUCs around 0.7646 and 0.6407, respectively. These results were the same for each repetition run.

## Discussion and limitations

The goal of our study was to assess the predictive power of radiomics w.r.t. mortality and troponin for pulmonary embolism patients.

Accuracy, F1-scores and AUCs show similar values. This shows a good balance between precision and recall as well as an overall correctness of the model’s prediction up to 97%. The analysis therefore seems to be stable over different metric calculations, which excludes possible data leakage, overfitting and data bias. This stability can also be seen in the radar charts as shown in Figs. 4 and 5. Completely overlaying shapes in radar charts would indicate a consistent performance of the model over all metrics. Our metrics lines are close to each other with small variations, which is common because of variability in the data and the model predictions. It can indicate that the model doesn’t experience overfitting and represent a good performance over different criteria.

The values of $run_\mathrm{{{mort}}}$ are higher than of $run_\mathrm{{{trop}}}$ suggesting a slightly better predictability of the patients 30-day mortality (>0.98$-$5.8%). With values over 91% for $run_\mathrm{{{trop}}}$, a positive correlation between troponin and the extracted features was still demonstrated.

When comparing the 2D and 3D results, higher values are achieved for the metrics of the 2D masks in $run_\mathrm{{{trop}}}$ (>1.82–2%) and in $run_\mathrm{{{mort}}}$ for the 3D masks (>2.5$-$2.81%). This can also be seen in the previously mentioned radar charts as shown in Fig. 4 and 5. The 3D masks, compared to the 2D ones, are using shape features for the classification task. So it seems that these features are leading to better results in the prediction of the mortality, but not of troponin. Based on this outcome, the extracted and reduced three-dimensional features in this analysis appear to not be of high importance for the biomarker troponin.

Even though the same data are used for the repetition runs, standard deviation ranges smaller than 0.018 were obtained. These values could be explained due to the nature of the feature selection methods, which can vary in the reduced features even if the data stays the same. If the selected features change, the classification models perform differently, because other features than before are used as an input. Methods like NN also comprise a certain randomness by utilizing random initialization of weights during their algorithm. If we look into the differences between $run_\mathrm{{{mort}}}$ and $run_\mathrm{{{trop}}}$, the standard deviations for troponin are slightly higher (>0.0093$-$0.0112 for 3D masks). The analysis seems to be more stable for $run_\mathrm{{{mort}}}$. In the comparison of 2D and 3D analysis, the 3D results vary more in their values (>0.0065$-$0.0179). This could indicate that the 2D analysis in general is more reliable during reproducibility runs.

Compared to previous studies of APE and radiomics, we were able to achieve higher metric results. Surov et al. used the same dataset to quantify the predictability of radiomics features. These were contrary to ours extracted from the epicardial adipose tissue (EAT) of the patient’s lung. With 2D masks and other pre-processing and validation methods, they achieved accuracies of 0.88, an AUC of 0.776 (Cl. 0.671$-$0.881) and an AUC for an independent external validation cohort of 0.721 (0.633$-$0.808) [13]. Our accuracies and AUCs for $run_\mathrm{{{mort}}}$ are slightly higher and present a smaller deviation in their reproducibility (standard deviation < 0.0001). We utilized different pre-processing, including data balancing, as well as feature selection and classification methods and also analyzed the pulmonary artery thrombus, 2D and 3D representation, instead of the EAT. In contrast, we also addressed troponin predictability additionally. Other studies show similar results to Surov et al. Wang et al., for example, evaluated the thrombus area and achieved with 4 reduced features an AUC of 0.76 with also a higher range of values (0.67$-$0.84) [8].

We also see some similarities regarding the best performing classification methods even if many studies don’t apply all methods which we covered. For $run_\mathrm{{{mort}}}$, we identified SVM, XGB and EBT as well as KNN and LRC as the best classification methods. In general, SVM is said to be very successful regarding radiomics tasks [27] besides NN which demonstrated good results more often in recent years [28]. The previous mentioned paper of Wang et al. defined RF as the best and LRC as the second best method [8]. Deng et al. used lung graphs for the radiomics analysis of APE patients and found XGB to be the best followed by RF, LRC and SVM [29]. XGB was also determined as the best classification method by Shahzadi et al. who looked specifically into the image features of the skeletal muscles and intramuscular adipose tissues of APE patients [30]. Most of these findings were also shown by our analysis.

For the feature selection methods, LASSO and Chi2 are often observed to result in better predictions [31]. We found RF, LOR, and CARTC followed by ANOVA and LIR to be the best methods for our analysis. LASSO was not considered in our feature reduction, but Chi2 resulted often in the highest score with its number of occurrences close to the best achieving methods overall.

We compared our results with the predictive power of the RV/LV ratio as well. The scientific papers we evaluated for this analysis were able to achieve an AUC up to 0.8 for the prediction of the severity of APE [32, 33]. The mortality prognosis we performed with the calculated RV/LV ratio of the patients was less for mortality and troponin (< 0.1589$-$0.29). So in general the radiomics approach lead to better results for our data. Furthermore, only around 40% of patients have a right ventricular dysfunction in general [34, 35], which makes the prediction with the RV/LV ratio for a universal dataset less practical compared to the radiomics analysis.

The features which were mostly selected as important were of the Firstorder and Shape class. Firstorder contains the basic information of an image and represents the distributions of the values of the individual voxels without considering spatial relations. Shape features describe geometric properties of the evaluated area [16]. The singular voxel information as well as the general shape (for 3D) hence appears to be of importance for our radiomics analysis. Especially the spread of the thrombus as well as how compact the shape is seems to have an influence on this study. GLCM, which portrays the probability that a voxel appears at a certain direction and distance, was also of relevance [36]. If we look at other studies, they also often had features of the Firstorder and GLCM class selected as significant [29].

Our study is limited by the size of the data set. Manual segmentation was very time-consuming and could include more than 400 manually drawn segmentations for a single dataset. In addition, image resolution or image artifacts as well as incomplete clinical parameters complicated curation of the datasets. To reduce the influence of possible artifacts caused by contrast agents in the Vena Cava, our database comprised thrombi at different parts in the lung arteries. In case of an thrombus next to the Vena Cava, there were also parts of the thrombi unaffected by any artifact. We expect that our findings are therefore generalizable. In the future, a larger database could further improve this issue including automatic segmentation of lung thrombus.

## Conclusion

In this study, we conducted a manual segmentation of pulmonary embolism patients in order to extract radiomic features for prognosis of patient outcome and correlation with troponin. The resulting models yield up to 96.9% for accuracy, 0.97 for the F1-score and 0.9686 for the AUC. The analysis for the 3D masks and mortality as target variable appears to achieve the best results. We observed classification and feature selection methods which were also found out by other studies to perform the best. Based on our results, voxel and shape characteristics seem to have an importance when it comes to the radiomics analysis of the pulmonary artery thrombus area. For troponin, which wasn’t addressed in the radiomics context for APE yet, we could prove that its values correlate with radiomics features. We also demonstrated that radiomics feature of the pulmonary artery thrombus area could be used to predict mortality.
